# 0894. Time course of VILI development: a CT scan study

**DOI:** 10.1186/2197-425X-2-S1-O20

**Published:** 2014-09-26

**Authors:** C Chiurazzi, M Gotti, M Amini, C Rovati, I Algieri, M Brioni, A Cammaroto, C Bacile di Castiglione, K Nikolla, C Montaruli, S Luoni, B Comini, G Rossignoli, G Conte, T Langer, M Cressoni, L Gattinoni

**Affiliations:** Università degli Studi di Milano, Dipartimento di Fisiopatologia Medica e dei Trapianti, Milano, Italy; Fondazione IRCCS Ca' Granda - Ospedale Maggiore Policlinico, Milano, Italy

## Introduction

Mechanical Ventilation at high tidal volumes and pressures induces in lung oedema which is undistinguishable from ARDS. It is possible that pleura, bronchi and vessels act as a natural stress raiser playing as local stress multipliers. [1]

## Objectives

To study the development of VILI with CT scan and determine where and when the first lesions appear and to follow their development in time.

## Methods

Piglets were instrumented and ventilated with TV of corresponding to a strain (TV/FRC) >2.5. The whole study was performed in an animal CT scan facilities equipped as an ICU. Every three hours a CT scan was performed and data on respiratory mechanics were collected. CT scans were analysed and lesions were defined as clearly defined regions of poorly/not inflated tissue (-500/+100 HU) with a minimal diameter of 6 mm non present in the previous CT scan image. Lung weight was computed from the CT scan data.

## Results

**Figure 1:**
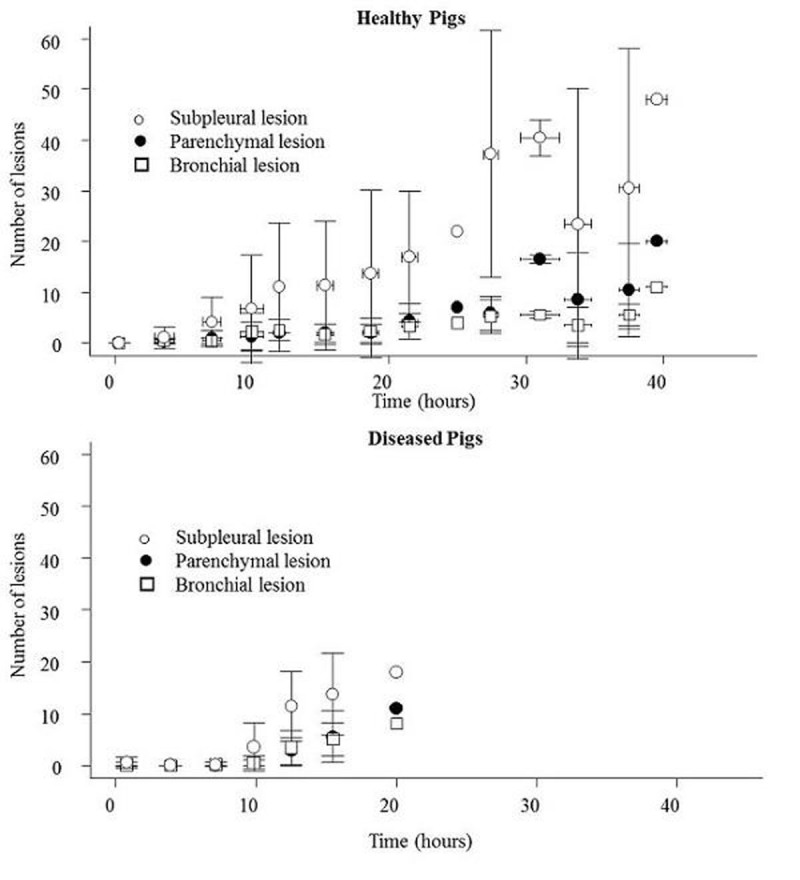
VILI Lesion Time Course.

We studied 11 swine (22±5 kg), 5 healthy and 6 with basal densities which did not regain inflation after a recruitment manoeuvre. As shown in Figure 1 most the CT scan lesions were subpleural. The first CT scan lesions appeared after a median of 7 hours [4.5-12] in healthy pigs and after a median of 9 hours [6.5-11.5] in diseased ones (p=0.88 healthy vs diseased) while changes in lung mechanics developed after 20 [13 - 23] hours (p=0.04 vs time of development of CT scan lesions) and oxygenation impairment, defined as PaO2FiO2 < 200, after 25 [14 - 36] hours (p=0.003 vs time of development of CT scan lesions) while widespread lung oedema defined as presence of infiltrations in all lung fields at CT scan imaging developed after 18 [12 - 32] hours (p=0.02 vs time of development of CT scan lesions).

## Conclusions

Oxygenation and respiratory mechanics impairment is evident when relevant lung oedema develops. Most of the first CT scan lesions are located in lung regions which act as stress raisers and precede alterations in gas exchange and respiratory mechanics.
